# Palpation sensing for robotic-assisted surgery

**DOI:** 10.1007/s00132-025-04753-1

**Published:** 2025-12-10

**Authors:** Michael Friebe

**Affiliations:** 1 Paul-Schürholz-Str. 7, 45657 Recklinghausen, Germany; 2 Faculty of Computer Science, HealthTech Innovation Laboratory, AGH University of Krakow, Krakow, Poland

**Keywords:** Robotic-assisted surgery, Haptic feedback, Minimally invasive surgery, Artificial intelligence, Tactile sensing, Roboterassistierte Chirurgie, Haptisches Feedback, Minimal-invasive Chirurgie, Künstliche Intelligenz, Taktile Sensorik

## Abstract

Palpation and haptic feedback are vital for improving surgical precision, safety and decision-making in robotic-assisted surgery (RAS). Current RAS systems largely rely on visual input, lacking tactile feedback and autonomy, which increases training demands and limits scalability. The absence of touch impairs tissue characterization, complicates diagnostics and elevates the risk of unintended damage. Integrating haptics, through force sensors, tactile interfaces and emerging audio-based tools, has shown promise in restoring a sense of touch, particularly when enhanced by artificial intelligence and multimodal feedback systems. These technologies will enable interpretation of real-time data, improved tissue discrimination and reduced application of force, especially valuable in minimally invasive procedures. While progress has been made challenges remain in sensor miniaturization, biocompatibility and system integration; however, achieving semiautonomous and fully autonomous RAS requires intelligent sensing platforms combined with AI-driven analytics, and feedback mechanisms that approach human tactile perception. As tactile simulation technologies evolve, future surgical robots will operate with greater autonomy, improved accuracy and broader global accessibility. The field is moving toward a new paradigm: surgical robots as intelligent, adaptive systems capable of performing or assisting procedures collaboratively or independently, using real-time sensing and control to optimize outcomes and reduce reliance on human expertise.

## Abstract (de)

Palpation und haptisches Feedback sind für die Verbesserung der chirurgischen Präzision, Sicherheit und Entscheidungsfindung in der robotergestützten Chirurgie (RAS) von entscheidender Bedeutung. Aktuelle RAS-Systeme stützen sich weitgehend auf visuelle Eingaben, wobei taktiles Feedback und Autonomie fehlen, was den Schulungsaufwand erhöht und die Skalierbarkeit einschränkt. Das Fehlen von Tastsinn beeinträchtigt die Gewebecharakterisierung, erschwert die Diagnose und erhöht das Risiko unbeabsichtigter Schäden. Die Integration von Haptik durch Kraftsensoren, taktile Schnittstellen und neue audiobasierte Tools hat sich als vielversprechend für die Wiederherstellung des Tastsinns erwiesen, insbesondere wenn sie durch künstliche Intelligenz und multimodale Feedback-Systeme ergänzt wird. Diese Technologien ermöglichen die Interpretation von Daten in Echtzeit, eine verbesserte Gewebediskriminierung und eine geringere Krafteinwirkung, was insbesondere bei minimal-invasiven Eingriffen von großem Wert ist. Trotz der erzielten Fortschritte bestehen weiterhin Herausforderungen in Bezug auf die Miniaturisierung der Sensoren, die Biokompatibilität und die Systemintegration. Um halb- und vollautonome RAS zu realisieren, sind intelligente Sensorplattformen erforderlich, die jedoch mit KI-gestützten Analysen und Rückkopplungsmechanismen kombiniert werden müssen, die sich der menschlichen Tastwahrnehmung annähern. Mit der Weiterentwicklung der taktilen Simulationstechnologien werden zukünftige Operationsroboter mit größerer Autonomie, verbesserter Genauigkeit und breiterer globaler Zugänglichkeit arbeiten. Der Bereich bewegt sich auf ein neues Paradigma zu: Operationsroboter als intelligente, adaptive Systeme, die in der Lage sind, Eingriffe gemeinsam oder unabhängig durchzuführen oder zu unterstützen, wobei sie Echtzeit-Sensorik und -Steuerung nutzen, um die Ergebnisse zu optimieren und die Abhängigkeit von menschlichem Fachwissen zu verringern.

## Need for palpation in robotic surgery—Introduction

Palpation and haptic feedback are critical not only for improving surgical precision and outcomes but also for enabling predictive models, aiding surgeon decision-making and supporting the development of semiautonomous and fully autonomous RAS systems [1, 2].

Across a colorectal, gut, biliary, hepatic, cardiac and also orthopedic context, the lack of palpation/haptic feedback in RAS manifests through the need for higher forces, more errors and longer task times, while controlled data show that restoring haptics improves force control, accuracy, speed, and success. There are clear, quantifiable gaps that are most evident in procedures demanding fine tissue discrimination and delicate traction [3].

Ultimate goal is to develop robotic surgery systems that can function independently of human experts

Currently, RAS systems offer minimal autonomy and lack tactile feedback, relying almost entirely on visual input from integrated cameras. This necessitates extensive preoperative training, making educational tools for minimally invasive surgery and therapy (MIS/MIT) essential.

The ultimate goal must be to develop intelligent, accessible, predictive, and affordable robotic surgery systems that can function independently of human experts, delivering high-quality care globally.

Achieving this requires combining advanced sensors, integrated imaging and AI for real-time surgical assessment and prediction. Adding palpation and haptic sensing is a crucial step toward semiautonomous operation.

## Status quo and definitions

Palpation (Latin palpatio: to touch) is a tactile diagnostic technique where following visual inspection clinicians use their hands to assess tissue, organ, bone, joint or soft tissue conditions. It provides qualitative and limited quantitative data on temperature, moisture, tenderness, pulses, masses, swelling and deformities.

The absence of haptic feedback is particularly pronounced in RAS

The absence of haptic feedback is particularly pronounced in RAS where the physical separation between the surgeon and the patient exacerbates the lack of direct touch sensation.

Surgeons often compensate for the lack of tactile feedback in MIS/MIT or RAS by relying on visual cues from integrated cameras or external imaging and by investing substantial time in training; however, this approach can extend procedure times and increase complication risks compared to open surgery. Improved haptic feedback systems are therefore essential as they help surgeons to perceive mechanical properties more accurately by integrating visual and tactile information to enhance motor performance [4].

## Robotic surgery systems/robotic-assisted surgery (RAS)

Robotic surgery involves telemanipulated systems controlled directly by surgeons, primarily for minimally invasive procedures. These systems improve precision, dexterity and visualization, enabling smaller incisions, reduced blood loss and faster recovery compared to open or standard minimally invasive surgery [5, 6]; however, key limitations remain, especially in tissue characterization and palpation.

Surgeons traditionally (in open and MIS/MIT) rely on kinesthetic (force) and tactile (texture, compliance) feedback to assess tissues. Robotic systems enhance vision but eliminate these sensory cues [7].

Without palpation, distinguishing healthy from diseased tissue or detecting subtle pathological changes is more difficult. While advanced imaging helps, it cannot fully replicate the information provided by touch [8]. Modalities, such as X‑ray images, MRI and ultrasound can assist with localization and guidance but they also introduce added cost and complexity. Moreover, imaging often misses subtle issues, such as microtears in ligaments, tendons, or myofascia that palpation can detect, helping align the diagnosis with patient symptoms and physical findings [9].

Palpation sensation can provide information that may not even be visible on imaging

Palpation sensation can provide information that may not even be visible on imaging, such as subtle tenderness or small soft tissue abnormalities.

## Towards semiautonomous and autonomous RAS

Most current robotic systems are not autonomous, but are best described as robot-assisted, with the surgeon maintaining full control. A recent systematic review applying the levels of autonomy in surgical robotics (LASR) scale found [10] (see Table 1) that most systems currently on the market are level 1 and only very few operate in levels 2 and 3, with no systems in levels 4 and 5.

**Table 1:** Levels of autonomy in surgical robotics

Level	Description	Current clinical use
0	No autonomy—Manual tools, no robotic assistance	Not robotics, typical minimally invasive surgery approach
1	Robot assistance—Surgeon in full control, robot provides physical/cognitive aid	Most common
2	Task autonomy—Robot can autonomously perform specific tasks when instructed	Rare, emerging
3	Conditional autonomy—Robot can plan tasks, adjust actions with surgeon oversight	Very limited, advanced systems only
4	High autonomy—Robot plans and executes sequences with minimal human input	Not in clinical use
5	Full autonomy—Robot performs entire procedures independently	Not in clinical use

Achieving higher autonomy in surgical robotics requires more sensory data from the surgical site, real-time imaging and AI trained on complete procedures to predict outcomes based on current conditions.

Without more autonomous operation the controlling human operator will remain the limiting factor

Without more autonomous operation it will not be possible to scale the use of RAS systems as the trained and controlling human operator will remain the limiting factor.

Integrating additional sensors and AI enhances tissue discrimination and can support pathology detection by identifying differences in stiffness, elasticity and compliance. For instance, autonomous systems using force sensors and deep learning could estimate the depth of hard inclusions (e.g., tumors) in soft tissue, mimicking human palpation [11, 12].

Machine learning models trained on tactile and force data would help robots adapt to dynamic surgical conditions, such as tissue deformation. Surgeons report increased confidence and control with haptic feedback systems, indicating that semiautonomous systems must offer similar capabilities to earn clinical trust [13, 14].

Table 2 summarizes the current status of simulating palpation-related touch senses and their implementation in robotic technology.

**Table 2:** Aspects of touching and technologies implemented in RAS systems

Aspect of touch	Human sensation	Current robotic technologies	Status
Pressure	Highly nuanced	Basic force feedback, some tactile sensors	Partial replication
Vibration	Wide frequency range	Limited, some wearable devices	Emerging
Texture	Fine discrimination	Not widely replicated	Limited
Temperature	Higher or lower than normal	Not replicated	Absent
Proprioception	Natural, continuous	Kinesthetic feedback via sensors	Partial
Multidirectional feedback	Yes	Some wearable devices, limited in tools	Emerging

## Current issues with RAS systems

**Fig. 1:**
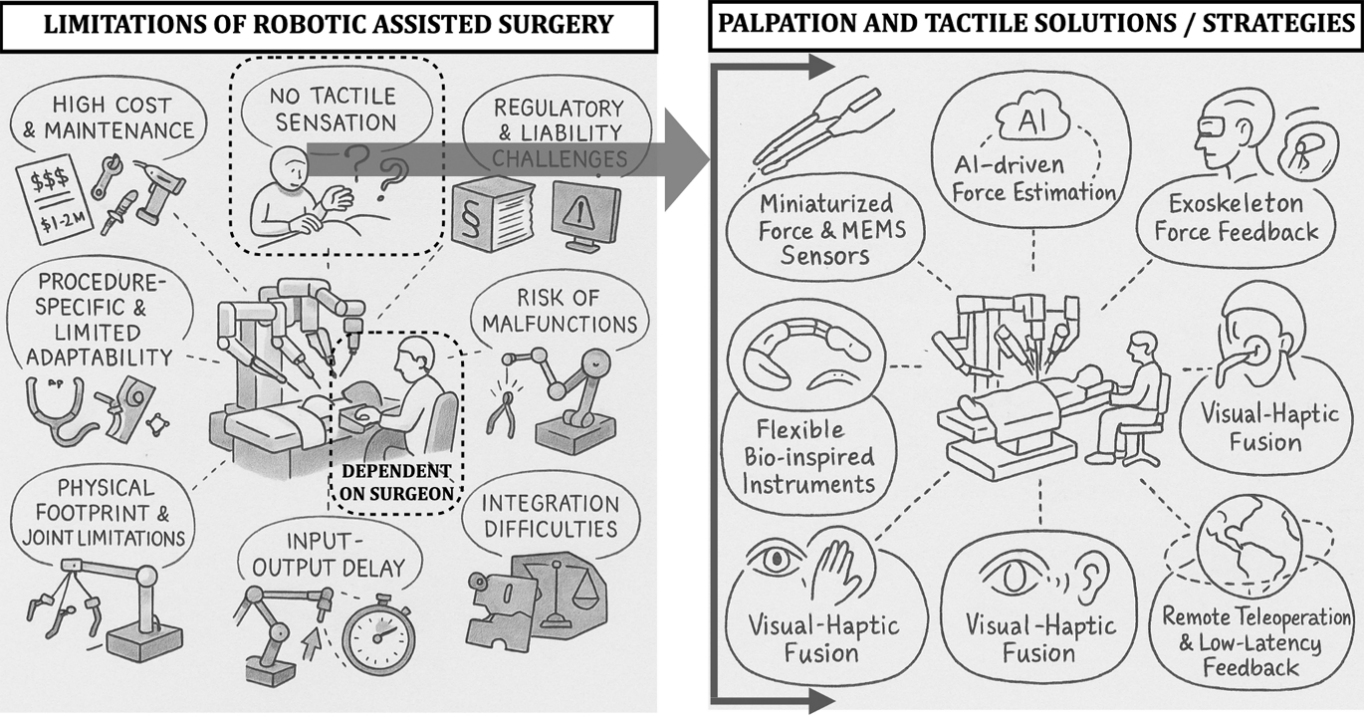
The limitations of robotic-assisted surgery (RAS) are shown on the left. Missing tactile sensation requires intense training to gain proficiency making the RAS very dependent on the surgeon. On the right some of the currently used solutions are listed that are explained in more detail in the manuscript. *MEMS* micro-electromechanical systems

There are several issues with current surgical robotics applications (see Table 3 and Fig. 1 left).

**Table 3:** The different issues with robotic assisted surgery (RAS) and its systems at the moment and as compared to more conventional minimally invasive surgery (MIS) approaches

RAS issues	DETAILS	Compared to more conventional MIS
1. High cost and economic barriers	High initial investment	Much cheaper in investment and operational cost and widely available
	High operational expenses	Tools can be reused after sterilization
	Not available on lower resource settings	
2. Lack of haptic feedback	Surgeons rely on visual cues at the moment	There is a direct connection through the tool between the surgeon and the tissue
	Force feedback sensors are provided in the newest generation	Training is relatively straightforward using simulators
	Requires long and extensive training	
	Can increase the risk of inadvertent damage	
3. Complex training and learning curve	Extensive training required to achieve proficiency	Every medical student undergoes minimally invasive surgery training
	Initial procedures can therefore take much longer and/or have higher risk of complications	Can easily be updated using simulators
4. Limited versatility and adaptability	Often systems are optimized for specific procedures	Systems and tools are much easier adaptable and dedicated surgical tools are widely available
	Difficult to adapt to changing situations during the surgery	
5. Technical and mechanical constraints	Large physical footprint with access restrictions	Requires relatively little space (endoscopic tower) in a surgery room and can be moved relatively easily
	Limited degree of freedom may restrict dexterity and restrict movement	
6. Dependence on human operator	Systems are primarily teleoperated	Surgeons ability and experience also very important but there are typically several experts available
	No autonomous system operation, relies heavily on the human operator	
7. Latency and responsiveness	Small but perceptible delay between operator input and tool movement	No latency
	Worse for remote telesurgery	No remote operation possible
8. Safety concerns	Risk of mechanical failure, software issues or instrument failure	Instrument failure can also happen, but typically replacement available immediately
	Emergency protocols needed	
9. Limited integration and interoperability	Difficulty integrating with other equipment	Only the endoscopic tower has to be integrated. Standard connection protocols (e.g. DICOM for image files)
	Imaging modalities	
	Health IT systems	
10. Regulatory and liability challenges	Complex regulatory approvals slow innovation	Innovations also need to be approved with similar timelines
	No real clarity on liability in case of malfunction

Surgeons rely solely on visual cues from a limited field-of-view camera and onsite imaging

While robotic surgery systems are expensive, complex and often limited to specific procedures, the core barrier to wider adoption is the extensive training required for human operators. There are simply not enough fully trained surgeons and they are concentrated in a few locations.

One major reason for the long training is the lack of palpation and haptic feedback: surgeons rely solely on visual cues from a limited field-of-view camera and in some cases additional onsite imaging.

Only when essential tissue information becomes available, either to the human operator or as sensor data for semiautonomous or fully autonomous systems, can training demands be reduced, decreasing reliance on human expertise. This could lead to broader deployment, increased competition and eventually lower system costs. While this economic logic may seem simplistic, the expansion and innovation of RAS systems will depend on greater automation and less dependence on human experts.

Another current limitation is the lengthy set-up and preparation time for RAS. In contrast, conventional MIS often achieves similar or faster outcomes [15].

Scientific evidence does not yet show a broad clinical advantage of RAS over conventional laparoscopic methods [16]. While it offers clear benefits in specific areas, such as urological and gynecological procedures [17], most surgeries show equivalent or only marginally better results, which often do not justify the higher costs (see Table 4, last column).

Randomized studies have not demonstrated a clear overall advantage using RAS over MIS/MIT

Randomized studies have not demonstrated a clear overall advantage using RAS over MIS/MIT, though evidence is growing for benefits in select procedures.

**Table 4:** Haptic and palpation solution ideas and technologies that are currently being researched

Haptic feedback and palpation sensation technologies	Details	Drawback/comment
1. Force sensor integration	Integrating miniaturized force and tactile sensors into surgical instruments to measure real-time tissue resistance and interaction forces	Dedicated tools needed
	Multimodal haptics: combine vibrotactile, kinesthetics and thermal feedback to simulate realistic tissue textures and resistance	Expensive
		May limit tool functionality
		Additional cables/electronics
2. Sensorized surgical instruments	MEMS and fiber-optic sensors: utilizing micro-electromechanical systems (MEMS) or optical fiber-based force sensors for precise and reliable measurement without significantly increasing instrument size	See above but less expensive and with lower negative effect on functionality
3. Wearable and tactile interfaces	Glove-based feedback devices: developing wearable gloves or fingertip sensors that translate measured tissue interactions into tactile sensations directly delivered to surgeons	Expensive
	Exoskeleton-based systems: employing exoskeleton devices providing force feedback and natural motion sensations directly to the surgeon’s hands or arms	Adds complexity
		Requires additional training
		Cumbersome
4. Virtual reality (VR) and augmented reality (AR) integration	Virtual haptic rendering: integrating VR environments with computational algorithms that simulate tissue interactions to offer realistic haptic sensations virtually	Simulation is always worse than actual data but in the combination with advanced sensor information and artificial intelligence and deep learning promising. Likely expensive and complex
	AR-enhanced force feedback: overlaying force interaction information directly onto surgical video streams, assisting surgeons visually when physical haptic sensations are unavailable	
5. Artificial intelligence (AI) and deep learning (DL)	Leveraging machine learning algorithms to predict tissue stiffness and force interactions based on visual data, motion tracking and instrument movements	Requires data input from the actual surgery (sensors, imaging). Very promising but still in research modus
	Deep learning-based tactile feedback generation: training neural networks to accurately infer and reproduce realistic tactile sensations from visual and robotic data	
6. Soft robotics	Flexible instrumentation: development of soft or compliant robotic instruments capable of passively adapting and providing intrinsic haptic cues based on deformation when contacting tissues	This is still research. No system close to actual clinical use. May still take some decades
	Bio-inspired robotics: designing instruments inspired by biological structures (e.g., tactile hairs, mechanoreceptors) to naturally sense and transmit haptic feedback	
7. Remote teleoperation improvements	Low-latency communication systems: Ensuring near-instantaneous feedback loops using improved communication protocols to preserve realistic haptic sensations, especially critical in remote surgery	No drawback. New G6 mobile networks promise to have little to no latency
8. Hybrid feedback approaches	Visual-haptic fusion: creating hybrid sensory modalities combining visual cues, audio feedback and tactile signals to reinforce surgeons’ spatial awareness and precision	More and more complex but will be the basis for more autonomy. Initially expensive with the hope to become cheaper
9. Clinical and experimental validation	In vivo and ex vivo testing: conducting extensive animal and cadaveric trials to fine-tune feedback mechanisms for surgical realism and clinical efficacy	Already done today and will remain a necessity for additional innovations
	Human factors research: collaborating closely with surgeons to iteratively evaluate and refine haptic feedback devices based on real-world clinical experiences and usability studies	
General recommendations	Miniaturization of sensing technologies without compromising durability	–
	Developing standards and protocols for haptic feedback fidelity in surgical robotics	
	Continued interdisciplinary collaboration involving engineers, surgeons, neuroscientists and human factors specialists	

## Sensor technologies and feedback concepts

**Fig. 2:**
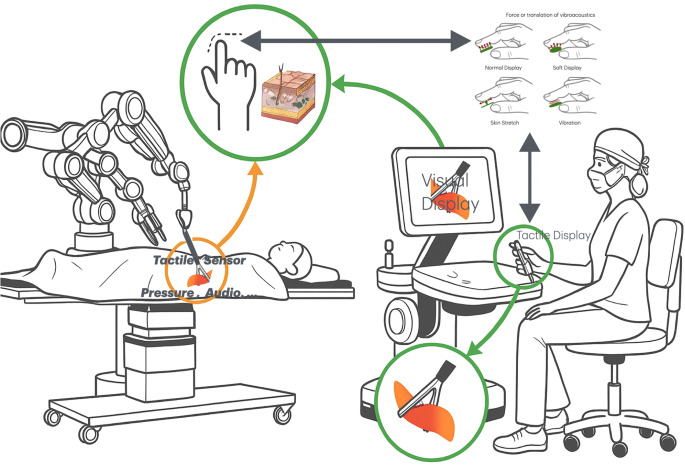
Sensors integrated in the surgical tool or attached to the shaft (e.g. force, pressure, audio, capacitive or others) obtain information about the tissue and translate it via a tactile display to the guiding hand or finger of the tool-controlling surgeon

The following technologies are increasingly being integrated into MIS tools. Due to their similar functionality and application they are also expected to become standard in RAS systems. Sensor integration typically occurs at the tool tip and in some cases along the shaft. The resulting data are conveyed to the surgeon through vibration, skin stretching or auditory feedback via tactile displays (see Fig. 2).

Promising approaches include:

Microminiature force sensors installed at the tips of surgical instruments (end-effectors), these advanced sensors measure interaction forces (push, pull, grip) between the tool and tissue. They provide real-time feedback to the surgeon, enabling perception of tissue resistance and consistency [18].

Proximally attached (outside the patient) audio sensors are an emerging concept where sensors on the tool shaft record vibroacoustic signals from tool-tissue interaction. These low-cost sensors can reveal tissue stiffness, surface properties and even detect perfusion sounds [2].

Tactile sensors replicate texture and firmness, helping differentiate soft from firm tissues critical for tumor detection or navigating delicate anatomy [19]. When paired with depth cameras they enhance tissue deformation analysis [9]. Modern tactile sensors also include flexible elements capable of detecting shear forces with high sensitivity.

In MIS these technologies aim to restore the tool-tissue-surgeon connection, improving feedback, decision-making and outcomes [7]. As RAS lacks direct tactile interaction, more advanced feedback systems have emerged:

Active force feedback systems; systems like the da Vinci 5 (Intuitive, Sunnyvale, CA, USA) now include force feedback, enabling sensation of pressure, tension and resistance during dissection, retraction or suturing. This can reduce the force applied by up to 43%, increasing safety and precision [18].

Combined kinesthetic and tactile feedback: new training platforms integrate both force (kinesthetics) and texture/grip (tactile) feedback for a more immersive and realistic surgical simulation environment [20].

Multimodal feedback systems: some systems combine force feedback with visual and auditory cues, creating a richer, more intuitive interface to enhance control and awareness [19].

AI-augmented haptics: machine learning algorithms analyze tactile data to enhance haptic feedback. Artificial intelligence (AI) can suggest optimal force levels, identify potential issues, or automate specific actions based on feedback patterns [21].

Haptic feedback is anticipated to play a pivotal role in the enhancement of palpation techniques

As the field of robotic surgery continues to progress, the integration of advanced haptic feedback systems is anticipated to play a pivotal role in the enhancement of palpation techniques. The integration of machine learning and imaging technologies will additionally enhance robotic surgery by providing real-time guidance and analytics. Surgeons benefit from augmented reality (AR) and virtual reality (VR) technologies that deliver detailed real-time imaging to improve navigation and precision. Combined with high-resolution 3D imaging, these tools aid in visualizing anatomy and identifying tissue properties during minimally invasive procedures. Together, these innovations are restoring the “sense of touch” in robotic surgery, enhancing safety, precision and overall outcomes [18, 19, 22].

A major challenge is the integration and miniaturization of sensors in the tools or systems

A major challenge remains with respect to integration and miniaturization of sensors in the tools or systems.

## How close are current technologies to replicating human tactile sensation in surgery?

Robotic-assisted surgery has advanced in restoring aspects of tactile sensation but still falls short of replicating the full nuance of human touch in open surgery. Newer systems provide real-time data on grasping forces, tissue stiffness and thickness. For instance, “off-the-jaw” sensors now deliver objective tactile feedback, enhancing precision and safety in minimally invasive procedures [7, 9].

Some training and surgical platforms integrate both kinesthetic (force) and tactile (surface/grip) feedback, achieving up to 95% accuracy in force/torque detection and significantly improving simulation fidelity and surgical control. Compact wireless wearable devices now simulate tactile sensations, vibration, stretch, pressure and twisting, enabling programmable, nuanced feedback [23, 24].

Tactile feedback has been shown to reduce excessive force and tissue damage, particularly aiding novice surgeons in mastering delicate tasks more quickly [23, 25].

Human touch involves complex cues like pressure, vibration, texture, temperature, proprioception most of which are still absent in current systems [26, 27]. Many advanced haptic tools face integration challenges due to constraints on size, sterilization, and biocompatibility [32].

While some systems approach the precision of human hands, achieving a fully “transparent” experience, where surgeons feel direct tissue interaction, remains elusive. Future systems with greater autonomy may overcome this barrier.

There is the need to develop and adopt a universal standard for haptic feedback in surgical robotics

With many different approaches and growing competition there is also the need to develop and adopt a universal standard for haptic feedback in surgical robotics, and widespread clinical use of high-fidelity tactile feedback is still emerging.

## Conclusion

Integrating palpation and haptic feedback into robotic-assisted surgery (RAS) is essential for enhancing safety, precision and outcomes. Current RAS platforms rely heavily on visual input, limiting functionality and requiring highly trained operators, factors that constrain scalability and increase costs. The lack of tactile feedback impairs tissue characterization and raises the risk of inadvertent damage.

Despite advances in force sensors, tactile systems and AI-based support tools, challenges remain in miniaturization, integration, biocompatibility and real-time processing. Promising developments include proximally mounted audio sensors and robotic palpation tools, particularly when combined with AI for real-time interpretation [7, 28, 29].

Numerous emerging sensor technologies, although not detailed here, are poised to generate rich multimodal data that will further simulate palpation in the near future [30–36].

As these technologies will integrate to a more sophisticated haptic feedback and collaborative functionalities to enable the future oriented definition:

A surgical robot is an intelligent system capable of performing or assisting surgical procedures

A surgical robot is an intelligent, learning-enabled system capable of autonomously or collaboratively performing or assisting surgical procedures, using real-time data, adaptive control, and integrated sensing to optimize precision, safety and patient outcomes.

## Abbreviations

AI: Artificial intelligence
AR: Augmented reality
DL: Deep learning
LASR: Levels of autonomy in surgical robotics
MIS: Minimally invasive surgery
MIT: Minimally invasive therapy
MRI: Magnetic resonance imaging
RAS: Robotic-assisted surgery
VR: Virtual reality
